# Development of Spectral Disease Indices for ‘Flavescence Dorée’ Grapevine Disease Identification

**DOI:** 10.3390/s17122772

**Published:** 2017-11-29

**Authors:** Hania AL-Saddik, Jean-Claude Simon, Frederic Cointault

**Affiliations:** INRA, UMR 1347 Agroecology, 21000 Dijon, France; jean-claude.simon@agrosupdijon.fr (J.-C.S.); frederic.cointault@agrosupdijon.fr (F.C.)

**Keywords:** spectral analysis, feature selection, genetic algorithms, classification, vegetation indices, vineyard, diseases

## Abstract

Spectral measurements are employed in many precision agriculture applications, due to their ability to monitor the vegetation’s health state. Spectral vegetation indices are one of the main techniques currently used in remote sensing activities, since they are related to biophysical and biochemical crop variables. Moreover, they have been evaluated in some studies as potentially beneficial for detecting or differentiating crop diseases. Flavescence Dorée (FD) is an infectious, incurable disease of the grapevine that can produce severe yield losses and, hence, compromise the stability of the vineyards. The aim of this study was to develop specific spectral disease indices (SDIs) for the detection of FD disease in grapevines. Spectral signatures of healthy and diseased grapevine leaves were measured with a non-imaging spectro-radiometer at two infection severity levels. The most discriminating wavelengths were selected by a genetic algorithm (GA) feature selection tool, the Spectral Disease Indices (SDIs) are designed by exhaustively testing all possible combinations of wavelengths chosen. The best weighted combination of a single wavelength and a normalized difference is chosen to create the index. The SDIs are tested for their ability to differentiate healthy from diseased vine leaves and they are compared to some common set of Spectral Vegetation Indices (SVIs). It was demonstrated that using vegetation indices was, in general, better than using complete spectral data and that SDIs specifically designed for FD performed better than traditional SVIs in most of cases. The precision of the classification is higher than 90%. This study demonstrates that SDIs have the potential to improve disease detection, identification and monitoring in precision agriculture applications.

## 1. Introduction

Plant pathogens pose a major threat to crops and reduce yields worldwide [1]. Marks occurring due to two different infections can be quite similar. Also, patches resulting of the same infection do not appear the same way depending on the crop variety and surrounding conditions. Thus, identifying crop diseases based on symptomatology alone is a complicated and subjective task and is often not sufficient. Additional laboratory tests are usually required to confirm the visual diagnosis.

Since 2013, about half of the French vineyard (400,000 ha) is placed in compulsory control zone against the FD and its insect vector [2]. The FD has also spread to other southern European countries (Italy, Portugal, Serbia and Switzerland) where it induced serious yield losses [2,3] and was declared as a quarantine body by the European Union. FD disease may result in the deterioration of the European grape quality. When FD is identified, massive amounts of pesticides are applied to prevent the propagation of the infection. This implies other problems such as chemical pollution and soil contamination. In order to efficiently apply pesticides, detecting and mapping initial established symptoms of diseases seems crucial.

Traditional methods for damage identification in crops include, first, visual inspection, a subjective and time-consuming approach particularly when large fields are scouted; second, laboratory-based techniques (Enzyme Linked Immunosorbent Essay (ELISA) or Polymerase Chain Reaction (PCR)), laborious biological techniques that require a strictly defined protocol in order to give a reliable result. Conversely, recent Remote sensing (RS) technology is capable of continuously analyzing information acquired by a device placed at a distance from the phenomenon of interest. RS devices can be grouped into two categories: imaging and non-imaging devices. In the first category, we find Hyperspectral/Multispectral Imaging, Fluorescence Imaging, Thermal Imaging and RGB Imaging. In the second category, we have Visible/Near Infra-Red Spectroscopy, Thermal Spectroscopy and Fluorescence Spectroscopy. RS techniques were exploited in the agricultural field in general [4,5] and have shown great potential in crop monitoring and yield mapping by providing new techniques that can replace or enhance classical approaches used in cultivar management [6,7]. Published scientific literature on the use of RS techniques to detect diseases are numerous. Hyperspectral Imaging was used by [8] to detect fusarium infection and by [9] to identify pathogens and necrosis in sugar beet. The authors in [10], used Multispectral Imaging to detect Huanglongbing in citrus trees. The study [11] consisted on using near-range and aerial hyperspectral sensors to detect fungal diseases. Other researchers combined existing techniques, for example, in [12] hyperspectral reflectance and multi-spectral imaging techniques were used together for fungal diseases detection in arable crops. Spectral reflectance is a valid tool to monitor vegetation’s health status. In fact, infections induce changes in pigments [13,14], water content and tissue functionality. These changes often alter the spectral characteristics of leaves. The spectral reflectance from sugarcane infected by thrips (Fulmekiola serrata Kobus) was significantly different between several damage severity levels and the highest difference occurred in the red edge region [15]. Other researchers, such as in [16], also studied reflectance alteration to identify leaf blight in rice.

One way to analyze vegetative spectral modifications due to an infection is the use of spectral vegetation indices (SVIs), calculated as ratios of reflectance at different wavebands. Many researchers investigated the potential of SVIs in detecting diseases. For example, [17] studied some sugar beet fungal diseases, [18] analyzed basal stem rot infection in oil palm trees. Focusing mainly on grapevines, [19], analyzed the spectral reflectance of red-berried leaves infected by Grapevine Leaf Roll disease (GLD) and found a set of variables capable of detecting pathogen presence. Another study was conducted by [20], it identified a feature vector made of 11 indices, able to spectrally differentiate healthy from GLD infected data.

In the above-mentioned studies, the SVIs used are common ones, however, the impact of plant diseases on the physiology and phenology of plants, varies with the host-pathogen interaction and each disease may influence the spectral signature in a different way. Common SVIs are not disease-specific (or disease-dependent); hence, it seems beneficial to design special indices for each infection (SDIs), as these might simplify the disease detection by spectral sensors. SDIs, unlike general SVIs, are designed to identify a specific damage in plants. They couple information from parts of the electromagnetic spectra which are characteristic of an infection and not visible to the human eye. Therefore, they may have the potential to automate the disease detection procedure, not only replacing to some extent the pathologist but also predicting the presence of an infection before its symptoms become noticeable.

In our study, spectral reflectance was acquired under production conditions, directly in the field. Furthermore, unlike other research works, that tested only one variety, we took into consideration 4 different grapevine varieties (2 red-berried grapevine and 2 white-berried grapevine). Based on the above background, the main objectives of this paper were (i) to identify disease specific single wavelengths and wavelength differences based on a GA feature selection tool, (ii) to combine these specific wavelengths to spectral disease indices and (iii) to compare the accuracy of the developed indices with respect to common SVIs.

## 2. Materials and Methods

### 2.1. FD Grapevine Disease and Identification Tests

The distinction between different grapevine diseases is a complicated task [21]. In fact, various diseases can cause almost identical symptoms; also, different symptoms may appear due to the same virus depending on the grapevine variety. Furthermore, marks can be the result of a fusion of many infections affecting the plant at the same time. Some factors such as bad weather conditions, nutrient deficiencies, pollution and pesticides can produce expressions indistinguishable from those of diseases; moreover, the time of infection and the overall environment can affect the appearance of signs on leaves. Present in the national territory since the mid-twentieth century, FD disease is transmitted to the vine by the Scaphoideus Titanus leafhopper and is progressing regularly in France [22].

**Figure 1:**
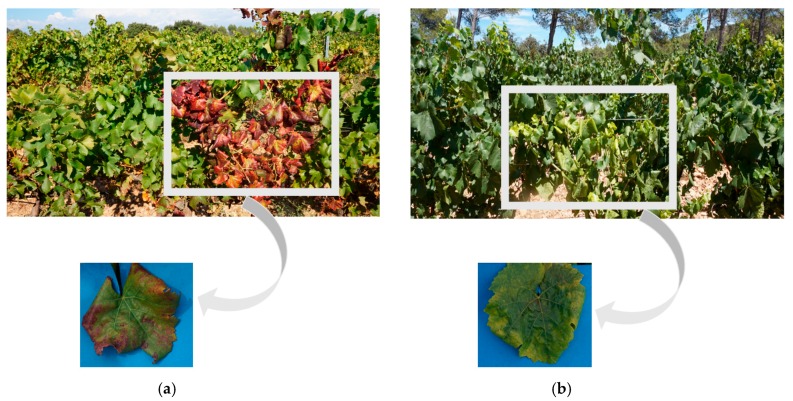
Some symptoms of FD on leaves: a red discoloration on a red grapevine variety (**a**) and a yellow discoloration on a white grapevine variety (**b**); windings of leaves can also be noticed.

Three symptoms must be present simultaneously (some are shown in Figure 1) and on the same branch to conclude the presence of FD [2,3,4,5,6,7,8,9,10,11,12,13,14,15,16,17,18,19,20,21,22,23]: the change in leaf coloration, the absence of lignification of the new shoots and the mortality of the inflorescences and the berries. For more details, [24] have reviewed in their paper the biology and the ecology of FD. In this study, we are assessing the possibility of FD detection based exclusively on foliar symptoms by employing spectral technology. The FD is difficult to detect because the characteristic symptoms usually appear at least one year after inoculation, not necessarily every year, nor on all the branches. In addition, grapevine varieties have different sensitivities with respect to FD, so the symptoms are not expressed the same fashion. Mainly, the discoloration of leaves varies according to the grape varieties (yellow for white-berried grapevines, red for red-berried grapevines). Other complications that arise when detecting FD is the similarity between its symptoms and those of other yellows of the vine such as ‘Bois Noir’ (BN); however, new chemical methods based on Polymerase Chain Reaction (PCR) are capable of detecting and differentiating BN from FD.

When FD is diagnosed in the field and in order to control the overall risk, uprooting contaminated vines regularly and applying pesticides to limit the population of leafhoppers, are the currently applied approaches.

### 2.2. Sampling Set-Up

**Figure 2:**
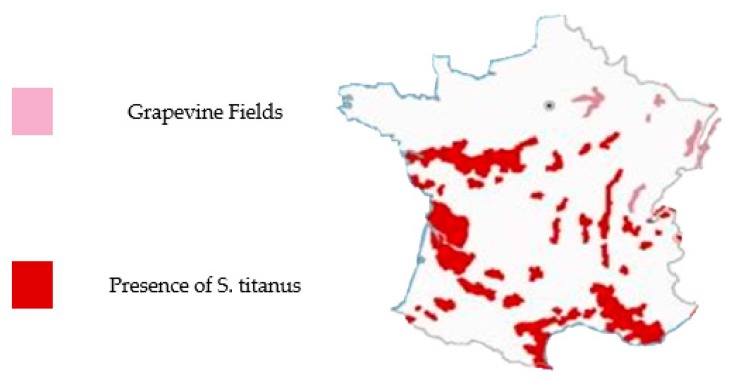
Vineyard distribution and S. titanus presence in France (from [24] modified).

In 2016, spectral signatures were considered from Provence-Alpes Côte d’Azur (PACA) French region (Figure 2). Two acquisition campaigns were conducted at the time in the PACA region. The first one took place on the 9th of August, where, symptoms affected only parts of the leaves. The second one took place on the 27th of September, where symptoms affected complete leaves.

Four grapevine varieties were tested, 2 red-berried ones (Marselan, Grenache) and 2 white-berried ones (Vermentino, Chardonnay). Red-berried fields were measured first in the morning from (10:00 to 12:00) and white-berried fields were measured next in the afternoon (14:00 to 16:00).

Measurements were performed on 2–4 leaves per grapevine and 2–4 measurements were made on each leaf. Four diseased and four healthy grapevines were considered for each grapevine variety. In total, there were 213 diseased and 201 healthy samples (63 Diseased Grenache and 64 Healthy Grenache; 63 Diseased Marselan and 64 Healthy Marselan; 47 Diseased Vermentino and 40 Healthy Vermentino, 42 Diseased Chardonnay and 34 Healthy Chardonnay). A range of healthy leaves of different ages was selected; however, for the infected leaves, a set is chosen in order to get a complete and representative range of FD symptoms. In order to ensure timely follow-up, the grapevines were located using a GPS and leaves were labeled. We tried to consider the same leaves during both acquisition campaigns but after testing them in August, leaves were not necessarily present in September; they either naturally fell or were cut by the winegrower. Thus, when the leaf was not found, we considered another candidate located on the same branch.

An inspector from the Regional Federation of Defense against Pests of PACA was there to confirm the presence of the disease and its severity stage. Furthermore, extra laboratory tests (PCR analysis) were done after the end of the acquisition campaigns to support the inspector’s claim.

### 2.3. Reflectance Measurements

Spectral reflectance is the ratio of incident to reflected radiant flux measured from a surface over a defined range of wavelengths. Spectral reflectance measurements from leaf surfaces, in this study, were acquired using a portable Spectro-radiometer (FieldSpec 3, Analytical Spectral Devices, Boulder, CO, USA). Measurements were made on each leaf using a plant probe, specially designed with a low power source, for sensible vegetation surfaces, leaving no observable damage. It has the advantage of reducing the effect of environmental light scattering to insure better measurement accuracy. Each sample data was taken every 1 nm from 350 nm to 2500 nm. This was the result of an interpolation performed by the software because the true spectral resolution of the instrument is about 3 nm at 700 nm wavelengths and about 10 nm at 1400 or longer wavelengths. Before starting the acquisitions, the spectro-radiometer was warmed up for a minimum of 20 min, then a calibration was performed to absolute reflectance using a Teflon calibration disk. The number of samples for Spectrum was set to 30, the number of samples for Dark Current and White Reference were set to 100. It took approximately four hours to complete the measurements directly in the field.

**Figure 3:**
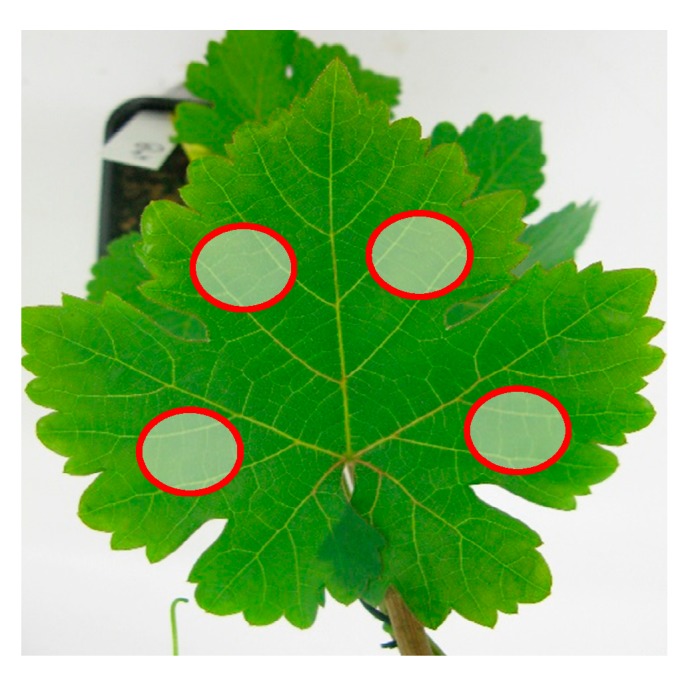
Locations of the measurements on a sample leaf.

Spectral measurements were taken from the same locations on leaves (shown in Figure 3) for both acquisition campaigns and one measurement is taken per location. The locations were chosen according to the disease that has tendency to start growing between the veins first. A range of 2–4 measurements is considered depending on the leaf surface with respect to the probe diameter. When the leaf is small, only 2 reflectance spectra from 2 locations are acquired and when the leaf is wide enough, 4 spectral tests are taken from all the 4 locations. The same procedure is applied for healthy leaves and infected leaves.

### 2.4. Spectral Data Analysis for Disease Detection

Each spectral measurement, acquired in this study, is the reflectance in a large number of contiguous narrow bands (350–2500 nm). Analyzing such high dimensional data is a complex and time-consuming task; therefore, reducing the dimensionality of the data, by selecting optimal wavebands, seems crucial. Techniques, such as SDIs, that uses only few spectral bands, are useful in the hyperspectral data analysis.

#### 2.4.1. Spectral Disease Vegetation Indices Development Based on GA Feature Selection (SDIs)

**Figure 4:**
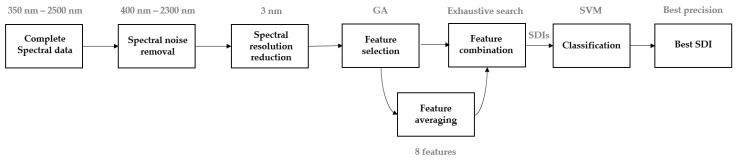
Systematical approach and development of SDIs from hyperspectral reflectance data.

In this section, the procedure is detailed from the acquisition of spectral signatures till the creation of disease-specific (or disease-dependent) indices. Figure 4 shows the approach that was adapted to compute and to evaluate SDIs.

**Figure 5:**
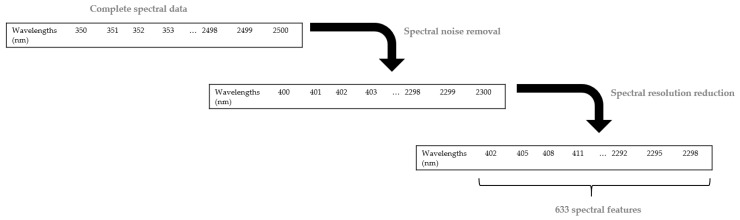
Noise removal and resolution reduction of spectral data.

After the acquisition of the spectral signatures of leaves from the field, we obtained a set of healthy and diseased observations ranging from 350 to 2500 nm, with a total of 2151 features or wavelengths. Since the spectral data were noisy at the extremities, values between 400 nm and 2100 nm were only considered and adopted, giving in total 1901 wavelengths. Following this, the spectral resolution was reduced by a factor of 3 due to high correlation between adjacent wavelengths. In consequence, only 633 wavelengths were considered for the rest of the analysis (Figure 5).

**Figure 6:**
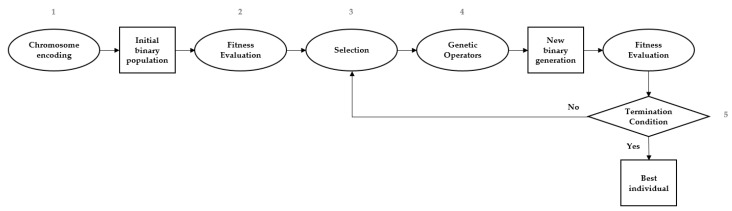
GA-Based Feature Selection.

From the modified set of observations obtained, the best wavelengths were chosen by applying a GA feature selection tool. Genetic Algorithms (GA) provides a valid tool for solving optimization and search problems; it imitates the natural human evolution process [25]. GA manipulates one population to produce a new one based on some genetic operators. The five important steps in GA [26] are: (1) chromosome encoding, (2) fitness evaluation, (3) selection mechanisms, (4) genetic operators and (5) criteria to stop the GA (Figure 6).

Human genetics vocabulary is often used in GA, chromosomes are the bit strings (individuals that form the population), gene is the feature [27]. In this study, a binary space is assumed: a gene value “1” indicates that the feature indexed by the “1” is chosen. Contrarily, (i.e., if it is 0), the feature is not chosen for evaluation. At the beginning, a matrix of dimension (Population size (300 samples) × Number of wavelengths (633 spectral features)) containing random binary digits is created, which forms the initial population. A fitness function evaluates the discriminative capacity of the population, made by chromosomes, each selecting a subset of features. In this work, the loss obtained by cross-validated SVM (Support Vector Machine) classification model is used. Individuals are ranked, based on the values reported by the fitness function; then, the Elite kids with the best fitness values, are selected to survive and are, hence, transferred to the next generation. The selection operation provides individuals for genetic cross-over and mutation; it ensures that the population is being constantly improved. Tournament Selection was used here due to its simplicity, speed and efficiency. Cross-over consists on combining two parent individuals to form children in the new generation. XOR operation is performed in this case since parent chromosomes are binary [28]. The number of new children produced due to the cross-over operator, is defined based on the cross-over fraction. Mutation is another genetic operator and induces a perturbation of chromosomes by applying a bit flipping procedure depending on the mutation probability. Mutation ensures genetic diversity, eliminating premature convergence. A uniform mutation is applied in this study. The number of new children produced due to the mutation operator, is defined by subtracting the population size from the number of elite children and the number of children obtained by cross-over. Each new generation, formed by GA, contains individuals from Elite kids, crossover kids and mutation kids [29]. The new population is evaluated again and the GA continues to evolve until the stopping condition is met. Two stopping conditions are applied in this study: Maximum Number of Generations and Stall Generation Limit. GA terminates if the average changes in the fitness values among the chromosomes over Stall Generation Limit generations is less than or equal to tolerance function. The goal is to insure genetic homogeneity. All the GA parameters used in our study are described in Table 1.

**Figure 7:**
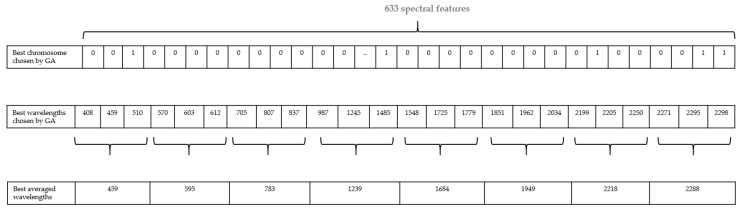
Example of feature averaging.

When GA terminates, one individual is chosen providing the convergence. This individual contains the optimal features, it is a binary set with “1” meaning that the feature at this specific index is considered. Since the initial population is randomly created, the number of selected wavelengths by the GA tool cannot be predicted and is function of the data, in fact, the GA keeps evolving until convergence and the number of features might be big. In order to reduce computational cost, we averaged the selected wavelengths chosen by GA to obtain only 8 wavelengths representative of the set (Figure 7). However, this feature averaging step is optional and all wavelengths selected by GA can be used in the feature combination step.

The indices to be developed aim at identifying a specific plant disease. Thus, a combination of a single wavelength and a normalized wavelength difference seemed suitable. A weighting factor for the single wavelength was determined and the possible weights were: −1, −0.5, 0.5 and 1. An exhaustive search of the best SDI is undertaken, combinations of an individual wavelength and a normalized wavelength difference are tested. Each combination of 3 wavelengths and a weighting factor forms an index (Equation (1)). When feature averaging is applied: 8 wavelengths × 7 wavelengths × 6 wavelengths × 4 weighting factors = 1344 possible combinations or SDIs were tested. The ideal case would be, again, to consider directly the wavelengths selected by GA with no averaging and evaluate all possible combinations. The indices were assessed for their classification ability using a 10-fold cross validation SVM model and the configuration providing the best classification precision is retained, this optimal configuration is the best SDI.

SDI = ab + (c + d)/(c − d)(1)

where a, c, d are wavelengths chosen from the pool of the 8 best averaged wavelengths (a ≠ c ≠ d) and b is the weighting factor.

#### 2.4.2. Common Spectral Vegetation Indices computation (SVIs)

In the context of vegetation status monitoring, identifying a specific disease or stress, can be done using spectral reflectance measurements. Discrimination between healthy and infected plants is performed based on some optimal wavelengths or a combination of wavelengths. The principal aim of SVIs is to highlight a certain property of the vegetation; they are combinations of reflectance at 2 or many wavelengths. Several vegetation indices have been proposed in the scientific literature, most of them relate the physiological status of crop to hyperspectral data through their correlation to biochemical constituents (chlorophyll, carotenoids, water, cellulose, lignin, dry matter…). Pigment-specific vegetation indices are, currently, an effective data analysis tool for disease discrimination. The ability to identify FD with vegetation indices, found in the literature, was tested in this segment. The classification accuracies of the NDVI, the PRI, the ARI, the SIPI, the mCAI, the PSSRa, PSSRb and PSSRc, the GM1 and GM2, the ZTM and the TCARI/OSAVI were compared to those obtained by SDIs (Table 2).

### 2.5. Classification

There are hundreds of classifiers in the literature and it is often difficult for researchers to choose an appropriate classifier for a certain application. The easiest approach that is used to address this issue is to try several classifiers and select the one having the highest accuracy. In this work, we selected only one classifier, the Support Vector Machines since it is one of the most widely used classifiers in the field and gave good performance in several applications [50,51].

#### 2.5.1. Support Vector Machines (SVM)

SVM is a supervised machine learning algorithm, mostly used to solve classification problems. It consists on defining a boundary (line/hyperplane) that best separates two classes [52]. The closest points to the boundary are called support vectors, the margin is the perpendicular distance calculated from the boundary to the support vectors. A maximal-margin classifier defines a hyperplane separating two classes and having the largest margin. However, a soft-margin classifier allows points to lie between the margins or on the wrong side of the plane. It is usually used when classes are not fully separable.

In practice, SVM are implemented using kernels. When applying non-linear Kernels (polynomial or radial), non-linear boundaries are created and the accuracy improves. Due to its flexibility, the Radial Basis Function (RBF) kernel is however the most used, so we employed it also in our study. One of the most known methods for fitting SVM is the Sequential Minimal Optimization (SMO) method. The concept and the applications of SVM are discussed in detail in [53].

#### 2.5.2. Data Configuration

**Figure 8:**
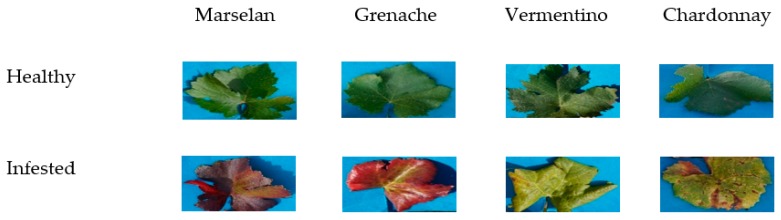
Configuration of analyzed data, infested against healthy groups for a binary classification.

In our study, we employed a binary classification involving only 2 classes: we considered the healthy group vs. the diseased group in total (medium infested measurements from the August acquisition campaign + high infested measurements from the September acquisition campaign). Since there are four grapevine varieties tested in this study (Marselan, Grenache, Vermentino and Chardonnay), it is possible to analyze the measurements of each variety alone, or measurements can be combined. Based on the grapevine color, we can analyze red types and white types; it is also feasible to combine all leaf measurements together (Figure 8).

## 3. Results

Spectroscopic and imaging techniques have demonstrated good potential in detecting disease and stress in crops. Currently, researchers tend to apply spectral vegetation indices (SVIs) to identify different plant diseases.

### 3.1. Reflectance Spectra of Diseased Grapevine Leaves

**Figure 9:**
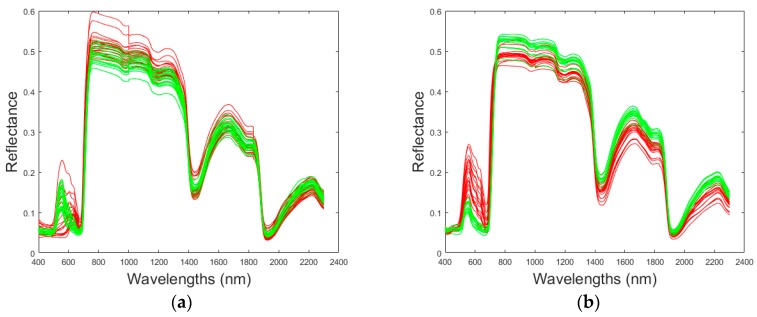
Reflectance of infected (Red) and healthy (green) leaves for Marselan (**a**) and Chardonnay grapevines (**b**).

When comparing spectral signatures of healthy and infected red/white berried leaves in Figure 9, obvious differences can be depicted, suggesting that the spectral response was affected by the infestation. For the Marselan variety (a red-berried variety), the healthy spectra were higher than the infested ones in the visible (VIS) region (mainly between 500–700 nm) but the opposite occurred in the region NIR (800–1300 nm) and in the IR region (>1300 nm). It seems like when the infestation arises, the spectral signature is lower in the VIS region and higher in the NIR-IR region, the same trend was also observed for the Grenache type (data not shown here). On the other hand, for the Chardonnay variety (a white berried variety), the healthy spectra were lower than the infested ones in the VIS region (mainly between 500–700 nm) but the opposite occurred in the region NIR (800–1300 nm) and in the IR region (>1300 nm). It seems like when the infestation occurs, the spectral signature is higher in the VIS region and lower in the NIR-IR region, the same trend was also observed for the Vermentino type (data not shown here). These changes prove that the spectral signature depends on the pathogen-host interaction. In other words, the grapevine variety does not show the same pattern when the same infestation occurs.

The mean value from a 10-fold cross-validation was reported for the classification; in this manner, all the data were taken into account and variances between different experiments under similar conditions were considered.

The model accuracy defined the percentage of testing set samples correctly classified and the False Negative Rate (FNR) defines the percentage of negative results that are, in fact, positive; in contrast, False Positive Rate (FPR) defines the percentage of positive results that are, in fact, negative. When plotting on a single graph, the FPR values on the abscissa and the TPR values on the ordinate, the resulting curve is called ROC (Receiver Operating Characteristic) curve, AUC (Area Under Curve) refers to the area under the curve. The advantage of using dimension reduction techniques based on GA will be demonstrated next.

### 3.2. No Dimension Reduction, Use of Complete Spectral Data

In this section, all spectral data are considered (400–2100 nm) in the analysis; this means that no dimension reduction method is applied in this case.

Table 3 presents the result of using complete spectra measurements from August (slightly infected leaves). The best classification accuracy is for Vermentino (93.75%) and the worst is for Marselan variety (70.97%). The Grenache and Chardonnay measurements gave similar precision (90.63%). What can be critical in disease diagnosis is probably the FNR, which means that a diseased case was claimed to be healthy. In general, the lower the FNR, the better the classifier is. Here, the best FNR was also for the Vermentino (6.67%). When considering combined measurements depending on the color of the grapevines, the observations of the White measurements were better than Red ones (92.19% > 87.3%).

Table 4 presents the result of using complete spectra measurements from September (highly infected leaves). The accuracy from September, in general, is better than that of August for all kinds of measurements. This seems logical, since symptoms at the end of the season become well established, diseased spectral reflectance are more influenced by the disease and can be more easily discriminated from healthy ones. White berried grapevines performed better that red-ones when each grapevine type is considered alone or combined (Marselan-Grenache 94.79–95.06% vs. Vermentino-Chardonnay 98.18–97.73%; Red 96.61% < White 98.99%). Furthermore, for White-berried leaves no FPR was reported. The hardest classification scenario is when all measurements were combined because observations from all grapevine types having different characteristics were put together. However, we obtained a satisfying SVM precision (96.01%) and a good AUC (0.99).

Table 5 presents the result of using complete spectra measurements from August in addition to those from September (slightly + highly infected leaves). Here the accuracy in general was better than considering moderately infected leaves (from August) but was less than using only highly infected leaves for the analysis (from September). The classification’s accuracy was above 92% for all cases: the best was for Chardonnay (97.37%), no FPR was found for this variety. When combining measurements was applied, similar results were found for Red, White and All configurations (around 95–96% of accuracy).

### 3.3. Dimension Reduction Using Vegetation Indices (SVIs)

In this section, the results of the classification using the common SVIs are presented. Only the best SVIs will be presented next, for more details refer to Table A1, Table A2 and Table A3.

Table 6 presents the result of calculating the best traditional SVI from August measurements (slightly infected leaves). The classification accuracies were satisfying (>90%) and they were more advantageous than using the complete spectra. No FNR was reported for Grenache leaves. The best SVIs were ARI, ZTM, TCARI/OSAVI. ARI is convenient for Red-grapevine varieties when considered individually or combined. ZTM behaved well for Vermentino and Chardonnay but when combined, TCARI/OSAVI performed better. This index was also robust when all observations are considered together (92.13%).

Table 7 presents the result of calculating the best traditional SVI from September measurements (highly infected leaves). In this case, all accuracies were enhanced with respect to those of August (>94%). When compared to using complete spectra, SVIs used less wavebands and gave better accuracies except for the case of mixing all measurements together (96.01% > 94.02%). The best results were associated with White-berried signatures and no FNR were found (97–98%). ARI, ZTM, GM1 and mCAI accomplished best precisions. Similar to the first acquisition campaign, the index ARI was interesting for the red-berried signatures. Moreover, ZTM was chosen for Vermentino and Chardonnay. However, when combined, GM1 was selected. mCAI was the most robust index when All measurements are mixed together (94.02%).

Table 8 presents the result of calculating the best traditional SVI from August measurements in addition to those from September (slightly + highly infected leaves). The performance was less than using only spectra with well-established disease marks from September. When compared to using complete spectra, Marselan, White-berried data were better or very similar to using complete spectra. However, for Grenache and mixed data the use of all wavelengths was more accurate. The best SVIs were ARI, GM1, ZTM and mCAI. ARI was again chosen to be the best SVI for classifying White grape leaves and was also selected when red grape leaves reflectance was tested (93.23%). GM1 was found to be interesting for Vermentino but ZTM was more convenient for Chardonnay. mCAI, like the above case, was the most robust index when all measurements are mixed together (88.41%).

### 3.4. Dimension Reduction Using Spectral Disease Indices (SDIs)

The discriminatory capacity of the best single wavelengths and wavelength differences chosen by the GA were tested. This data reduction procedure was the foundation for spectral disease index development. In this section, the results of the classification using the SDIs are presented.

Table 9 presents the result of calculating SDIs from August measurements (slightly infected leaves). A 100% success with no FNR in classifying individual grapevine measurements was obtained, except for Chardonnay. When combining observations, the results were also satisfying (precision > 94.44%). In general, better percentage was reached when applying the SDIs than using the complete spectra on one hand and applying conventional SVIs on the other hand.

Table 10 presents the result of calculating SDIs from September measurements (highly infected leaves). A 100% success with no FNR in classifying leaves measurements each variety at a time and when considering White-reflectance spectra together was obtained. In general, these accuracies are better than those corresponding to the first acquisition campaign. However, when the red varieties are grouped together, it seems that the ARI was better than the SDI (96.6% < 98.31%). When observations were combined, using complete spectra gave a slightly better result than the SDI (96.01% > 94.20%)

Table 11 presents the result of calculating SDIs from August measurements in addition to those from September (slightly + highly infected leaves). No FPR was present for White-berried data. In general, these accuracies are better than those corresponding to the first acquisition campaign. However, in accordance with the last case, when the red varieties are grouped together, it seems that the ARI was better than the SDI (92.03% < 93.23%). For the White-berried measurements and all mixed ones, it seems that using complete spectra is a bit more advantageous than the SDI but with more computation burden.

## 4. Discussion

SDI indices were put in place in this article to improve and simplify FD disease detection in grapevines based on hyperspectral data. At the beginning, the most significant wavebands from the VIS, Red-edge, NIR or SWIR (Short-Wave Infrared) needed to be selected.

Feature selection is often used in data pre-processing to identify relevant features having significance in the classification task. The results obtained in this study, confirmed the effectiveness of the GA algorithm in improving the robustness of the feature selection procedure. In fact, GA was able to reach a global optimum despite local peaks that might be caused by noise or interdependencies in the data set. This conclusion was also confirmed in other studies in different fields. In [54] GA selected the best subset of features for breast cancer diagnosis system. Furthermore, in [55] GA feature selection algorithm was applied for hand writing recognition. The complexity of the feature set was reduced using less features and achieved recognition rates similar to those reached when no feature selection is applied.

After the choice of certain wavebands by the GA tool was made, the SDIs were normalized in order to reduce the impact of change in lighting, land, crop variety or sensor specific effects. This helped producing more robust and more generalized indices. SDIs were more advantageous than complete spectra and SVIs in the beginning of the season (August measurements), hence, great promise for early detection of diseases. Using complete spectra was better for the case of combined measurements (August + September) for the Grenache, red-varieties and all data. However, the proposed indices proved high accuracy in general with the advantage of reducing data dimensionality by speeding up the disease detection. SDIs gave, in general, higher accuracy than SVIs but, the ARI index performed a bit better in September measurements for red varieties and all combined data than the corresponding SDIs. The ARI index is documented as a performant feature in many studies. The study [56] concluded that ARI had a persistent response to yellow rust disease at 4 out of 5 growth stages and mentioned that the ARI index was selected for diagnosis of yellow rust in other studies like the one conducted by [57]. Among the indices investigated in the research made by [58], only the ARI index could differentiate healthy from rust infected leaves. However, it was not capable of distinguishing stem rust from leaf rust pustules. In addition to the ARI index, ZTM, GM1, mCAI and TCARI/OSAVI were found to be the best SVIs in this study. The ratio of the TCARI and the OSAVI indices formed a good Chlorophyll estimator, this was done independently of Leaf Area Index (LAI) and illumination state. The ratio demonstrated good results not only in continuous closed crop canopies [49] but also in open tree canopy orchards [59]. Authors in [58] found that the TCARI index, was the only index capable of discriminating stem and leaf rust, among all others. Chlorophyll content is a potential indicator of vegetation stress because of its direct role in the photosynthesis process of light harvesting, initiation of electron transport [46], this was confirmed in our study as the ZTM vegetation index was chosen for white-berried data. Loss of chlorophyll in response to infestation by sap feeding insects like aphids [60] and leafhoppers [61] has been reported earlier. GM1 was also selected in this study; in fact, differences in reflectance between healthy and stressed vegetation due to changes in Chl; a, b levels have been detected previously in the green peak and along the red-edge spectral region of 690–750 nm [62]. The CAI index indicates exposed surfaces containing dried plant material [63]. Absorptions in the 2000 nm to 2200 nm range are sensitive to cellulose. It was stated in [64] that the CAI index is useful to monitor vegetation coverage for biomass estimation.

Many studies tended to manage pest occurrence in commercially important agricultural crops by designing new and adapted vegetation indices. Research [65] monitored damage by green bugs in wheat by using a hyperspectral spectrometer and a digital camera. They designed 2 indices based on possible band combinations and their correlation with the severity damage. Optimal bands were 509, 537, 572, 719, 747, 873, 901 nm. The study detailed in [66], on the other hand, used 2 or 3 narrow bands to design hyperspectral indices in order to assess severity grades of leafhopper in cotton. Two indices gave better results than traditional SVIs from literature and were consistent across tested fields. Interesting bands were: 550, 691, 715, 761, 1124 nm. In the research of [67], two indices were proposed and found to be capable of estimating leaf rust disease. The difficulty was to detect early symptoms due to the resemblance between spectral signatures between lightly infected areas and healthy ones. Based on this research, new indices performed better than other common SVIs. Optimal wavebands found were: 455, 605, 695 nm. (Mahlein et al., 2013) designed four SDIs and proved high specificity and sensitivity for identifying healthy leaves Cercospora leaf spot, Sugar beet rust and Powdery mildew. Detection of early symptoms of infection was the most difficult part in this research. Chosen bands varied from: 513, 520, 534, 570, 584, 698, 704, 724, 734 nm.

For August data, the majority of the selected bands were found in the NIR region; however, for September data, VIS bands were mostly selected since symptoms became more visually pronounced. In this case, bands from blue (450–520 nm), green (530–570 nm), red (580–700 nm) in the VIS were selected. This was in accordance with [19] the 2 maximum differences in the VIS region appeared at the green peak (550 nm) and in the red peak (680 nm) indicating less chlorophyll absorption in the infected leaves. Furthermore, changes in Cab levels were translated as modifications occurring over the spectral red edge region, this explains why many optimal bands were selected in the specific range of 690–750 nm. Reflectance near 700 nm was pointed out by [68] as an essential feature of green vegetation produced by an equilibrium between biochemical and biophysical plant characteristics. Since plant diseases influence the chlorophyll content of crop plants, increased reflectance around 700 nm can be a first but unspecific indicator to detect diseased crops. Many chosen bands were also mixed with the water absorption bands; the research conducted in [69], demonstrated that the sensitivity to water content was greatest in spectral bands centered at 1450, 1940 where water has its major absorption features.

It can be concluded, as seen from the tables, that the SDIs were dependent on the disease infestation level and the grapevine variety considered; the best wavelengths selected were different from one case to another. As a consequence, although the SDIs tested gave good results, there was no single best index for FD in all situations. In fact, the sensitivity of an index differs depending on the soil, the vegetation and the weather conditions. Therefore, no single index with the same spectral bands was found to be applicable to quantify FD in this research. SDIs were found to be interesting for precision agricultural applications; additional work will be needed in order to apply SDIs in practice. The proposed indices need to be tested on different varieties of grapevines before it can be effectively applied in precision farming. Our study enhances the ability to detect and map FD when foliar symptoms are becoming visible, hence, further tests need to be carried on hosts which do not provide any clear symptoms of infestation, to check if the computed SDIs are capable of predicting early the FD occurrence. Besides, suggested SDIs need to be tested in changing environments, scales and field conditions; other types of diseases must be also taken into consideration to investigate whether SDIs are capable of distinguishing FD from various infestations in general and BN in particular. At the end, it was proven, through this study that the development of indices based on spectral variations due to vegetation diseases, is feasible.

## 5. Conclusions and Perspectives

Plants display the occurrence of infections in a number of ways. RS is an effective way to detect crop diseases, based on the fact that a pest modifies the photosynthesis phenomenon and the physical structure of the plant, altering the absorption of light by the plant’s surface. The difference between spectral signatures of healthy and diseased plants can be the key to identify efficient wavelengths correlated with a specific disease. The transformation of reflectance into vegetation indices is a widely used technique to detect leaf contents (pigments, water, ...); nevertheless, these indices, based on only few wavelengths, showed potential for disease detection. Common vegetation indices are not yet capable of identifying a particular disease. In this article, a data analysis technique was exposed to design specific grapevine disease indices.

Our data set contains a large number of features, in order to reduce the cost and running time, as well as achieving an acceptably high recognition rate, we have selected the most useful ones by applying a GA feature selection tool. GA is one of the most advanced techniques used in the field of predictive analysis, it is computationally expensive but it performs better than common selection techniques and has the advantage of manipulating large data sets with no need for specific knowledge about the problem under study. After being selected, wavelengths are then combined to design the SDI. Depending on the disease severity, on one hand and on the grapevine variety on the other hand, a combination of a single and normalized wavelengths is required each time to correctly identify the FD.

Our study extracted some wavelengths bands sensible to FD occurrence at the leaf-scale. Based on these findings, it might be possible to conceive a multispectral camera, for example and mount the sensor on a movable platform to localize infection foci in a field. However, when going from considering leaves to examining a complete branch, or maybe the whole grapevine, some corrections need to be taken into account. Geometric and radiometric improvements capable to solve shadowing problems, branch structures and interfering reflectance from other surrounding objects are necessary. When applying a multispectral sensor, we will not only have spectral information but also spatial data. Available spatial data will enable adding advanced image processing algorithms to make the detection of FD more robust. Actually, two other FD symptoms cannot be detected spectrally, so, in order to better conclude the presence of the condition, additional pattern recognition algorithms can be integrated directly in the sensor to detect the absence of lignification and the berry mortality.

In accordance with other studies, we found that SDIs performed better than traditional SVIs. The advantages of using SDIs include the dimensionality reduction and the efficiency of computation and processing. The proposed method for SDIs development, in this article, can be transferred to hyperspectral data from different kinds of sensors, it can be used for other crops varieties and for different kinds of diseases or biotic and abiotic stress of crops.

## Figures and Tables

**Table 1:** Numerical values of different GA parameters considered in this study.

GA Parameter	Value
Population size (Number of Chromosomes)	300
Genome length (Number of genes/features)	300
Population type	Bit strings
Fitness Function	SVM-Based Classification Error
Number of generations	300
Stall generation limit	50
Crossover	Arithmetic
Crossover Probability	0.8
Mutation	Uniform Mutation
Mutation Probability	0.2
Selection scheme	Tournament of size 2
Elite Count	2

**Table 2:** Typical SVIs in literature and applied in this study.

Index Name	Formula	Association with Relevant Plant Pigment	Reference Example
Normalized Difference Vegetation Index (NDVI)	NDVI_705 = (R750 − R705)/(R750 + R705)	NDVI is a very typical index. Positive values suggest vegetated areas.	[30,31]
Photochemical Reflectance index (PRI)	PRI = (R570 − R531)/(R570 + R531)	PRI index is a function of the reflectance at the 531 nm, this reflectance is related to xanthophyll. When the xanthophyll activity is high, the light use efficiency is low, meaning a possible stress occurred.	[32,33,34]
Anthocyanin Reflectance Index (ARI)	ARI = (1/R550) − (1/R700)	ARI index is designed to estimate the stack of anthocyanin in senescing and stressed leaves.	[35]
The structure insensitive pigment index (SIPI)	SIPI = (R800 − R445)/(R800 + R680)	The SIPI index is responsive to the ratio of carotenoids to chlorophyll. It is very practical to use when the canopy structure or leaf area index are inconsistent.	[36,37]
Modified chlorophyll absorption integral (mCAI)	mCAI=(R545 + R752)/2 × (752 − 545) − (∑545 − 752 (1.158 × R))	The mCAI is sensitive to the chlorophyll content. It calculates the area between a straight line connecting two points (the green peak at 545 nm and 752 nm) and the curve itself	[38]
Pigment specific simple ratio chlorophyll a (PSSRa)	PSSRa = R800/R680	The pigment specific ratio indices were suggested to estimate the pigment’s content at the leaf level. Samples from trees at different senescence stages were studied aiming to empirically determine the best individual wavebands for pigment assessment (680 nm for chlorophyll a, 635 nm for chlorophyll b, 470 nm for the carotenoids).	[36,39,40]
Pigment specific simple ratio chlorophyll b (PSSRb)	PSSRb = R800/R635		[36,41,42]
Pigment specific simple ratio carotenoids (PSSRc)	PSSRc = R800/R470		[36,37,38,39,40,41,42]
Gitelson and Merzlyak 1 (GM1)	GM1 = R750/R550	GM1 and GM2 were created to measure the chlorophyll content in vegetation leaves.	[43,44]
Gitelson and Merzlyak 2 (GM2)	GM2 = R750/R700		[45]
Zarco-Tejada Miller (ZTM)	ZTM = R750/R710	ZTM is a Red edge index highly correlated to chlorophyll content. At the canopy level, it has the advantage of minimizing shadow effects.	[46,47]
Ratio of the Transformed Chlorophyll Absorption in Reflectance Index and Optimized Soil-Adjusted Vegetation Index (TCARI/OSAVI)	TCARI = 3 × ((R700 − R670) − 0.2 × (R700 − R550) × (R700/R670)) OSAVI = (1 + 0.16) × (R800 − R670)/(R800 + R670 + 0.16)	A combination of the Transformed Chlorophyll Absorption in Reflectance Index (TCARI) and the Optimized Soil-Adjusted Vegetation Index (OSAVI). It is sensitive to chlorophyll content variations and resistant to variations in Leaf Area Index (LAI) and underlying soil background effect.	[48,49]

**Table 3:** Results of using complete spectra in classifying different groups of spectral data acquired in the August acquisition campaign (Severity of infestation = 1).

Grapevine Variety	Accuracy (%)	FNR (%)	FPR (%)	AUC
Marselan	70.97	27.27	30.00	0.82
Grenache	90.63	7.14	11.11	0.73
Vermentino	93.75	6.67	5.88	0.93
Chardonnay	90.63	12.50	6.25	0.95
Red	87.30	7.69	16.22	0.96
White	92.19	9.38	6.25	0.93
All	88.19	13.85	9.68	0.95

**Table 4:** Results of using complete spectra in classifying different groups of spectral data acquired in the September acquisition campaign (Severity of infestation = 2).

Grapevine Variety	Accuracy (%)	FNR (%)	FPR (%)	AUC
Marselan	94.79	4.35	6.00	0.99
Grenache	95.06	2.38	7.69	0.97
Vermentino	98.18	3.23	0.00	0.96
Chardonnay	97.73	3.85	0.00	0.96
Red	96.61	2.22	4.60	0.99
White	98.99	1.75	0.00	0.93
All	96.01	2.08	6.06	0.99

**Table 5:** Results of using complete spectra in classifying different groups of spectral data acquired in the August and September acquisition campaigns (Severity of infestation = 1 & 2).

Grapevine Variety	Accuracy (%)	FNR (%)	FPR (%)	AUC
Marselan	92.91	9.23	4.84	0.98
Grenache	96.77	1.72	4.55	0.89
Vermentino	96.55	2.22	4.76	0.97
Chardonnay	97.37	4.65	0.00	0.97
Red	96.41	3.28	3.88	0.99
White	95.09	5.56	4.11	0.93
All	95.65	1.98	6.60	0.99

**Table 6:** Results of using the best SVIs in classifying different groups of spectral data acquired in the August acquisition campaign (Severity of infestation = 1).

Grapevine Variety	Accuracy (%)	FNR (%)	FPR (%)	AUC	Best SVIs
Marselan	90.32	11.11	7.69	0.94	PRI-ARI
Grenache	96.88	0.00	6.25	1.00	ARI
Vermentino	93.75	5.88	6.67	0.94	ZTM
Chardonnay	90.63	11.11	7.14	0.91	NDVI-ZTM
Red	95.24	5.88	3.45	0.99	ARI
White	92.19	11.11	3.57	0.92	TCARI/OSAVI
All	92.13	13.51	1.89	0.92	TCARI/OSAVI

**Table 7:** Results of using the best SVIs in classifying different groups of spectral data acquired in the September acquisition campaign (Severity of infestation = 2).

Grapevine Variety	Accuracy (%)	FNR (%)	FPR (%)	AUC	Best SVIs
Marselan	96.88	4.00	2.17	0.9805	ARI
Grenache	97.53	2.70	2.27	0.9988	ARI
Vermentino	98.18	0.00	3.23	0.9613	NDVI-mCAI-PSSRb-ZTM
Chardonnay	97.73	0.00	3.85	0.9579	NDVI-mCAI-PSSRb-GM1-GM2-ZTM-TCARI/OSAVI
Red	98.31	2.33	1.10	0.9991	ARI
White	98.99	0.00	1.75	0.9792	ARI-GM1
All	94.20	7.52	2.80	0.9540	mCAI

**Table 8:** Results of using the best SVIs in classifying different groups of spectral data acquired in the August and the September acquisition campaigns (Severity of infestation = 1 & 2).

Grapevine Variety	Accuracy (%)	FNR (%)	FPR (%)	AUC	Best SVIs
Marselan	93.70	8.70	3.45	0.9824	ARI
Grenache	91.13	13.70	1.96	0.9753	ARI
Vermentino	95.40	6.98	2.27	0.9730	GM1
Chardonnay	96.05	5.56	2.50	0.9672	GM2-ZTM
Red	93.23	11.19	0.93	0.9888	ARI
White	95.09	8.64	1.22	0.9433	ARI
All	88.41	16.96	5.98	0.9184	mCAI

**Table 9:** Results of using SDIs in classifying different groups of spectral data acquired in the August acquisition campaign (Severity of infestation = 1).

Grapevine Variety	Accuracy (%)	FNR (%)	FPR (%)	AUC	SDIs			
					a	c	d	b
Marselan	100.00	0.00	0.00	1.00	702	957	2133	1
Grenache	100.00	0.00	0.00	1.00	861	2094	921	−1
Vermentino	100.00	0.00	0.00	1.00	735	2097	1029	0.5
Chardonnay	96.87	5.88	0.00	0.93	543	876	1380	1
Red	95.23	6.06	3.33	0.97	1506	2214	507	0.5
White	96.87	3.12	3.12	0.98	792	2151	654	−0.5
All	94.48	6.15	4.83	0.98	1401	2205	501	−0.5

**Table 10:** Results of using SDIs in classifying different groups of spectral data acquired in the September acquisition campaign (Severity of infestation = 2).

Grapevine Variety	Accuracy (%)	FNR (%)	FPR (%)	AUC	SDIs			
					a	c	d	b
Marselan	100.00	0.00	0.00	1.00	546	708	597	−1
Grenache	100.00	0.00	0.00	1.00	528	540	1383	1
Vermentino	100.00	0.00	0.00	1.00	708	1656	1755	0.5
Chardonnay	100.00	0.00	0.00	1.00	462	570	888	−1
Red	96.61	5.68	1.12	0.97	738	1650	573	1
White	100.00	0.00	0.00	1.00	726	2166	927	1
All	94.20	8.95	2.81	0.98	498	675	1581	−1

**Table 11:** Results of using SDIs in classifying different groups of spectral data acquired in the August and the September acquisition campaigns (Severity of infestation = 1 & 2).

Grapevine Variety	Accuracy (%)	FNR (%)	FPR (%)	AUC	SDIs			
					a	c	d	b
Marselan	93.70	7.57	4.91	0.95	1653	2181	687	−1
Grenache	95.16	7.46	1.75	0.96	651	1944	549	1
Vermentino	96.55	6.97	0.00	0.97	687	1908	762	−0.5
Chardonnay	98.68	2.85	0.00	0.98	486	558	966	−0.5
Red	92.03	7.87	8.06	0.95	1725	2226	1485	−0.5
White	98.15	3.89	0.00	0.98	714	1404	936	0.5
All	89.37	12.79	8.37	0.92	1770	2208	2019	−0.5

## Abbreviations

The following abbreviations are used in this manuscript:

FD	‘Flavescence Dorée’
BN	‘Bois Noir’
GA	Genetic Algorithm
SVI	Spectral Vegetation Index
SDI	Spectral Disease Index
VIS	Visible
NIR	Near-Infra red
PACA	Provence Alpes Côte d’Azur
SVM	Support Vector Machines
FPR	False Positive Rate
FNR	False Negative Rate
ROC	Receiver Operating Characteristic
AUC	Area Under the ROC
NDVI	Normalized Difference Vegetation Index
PRI	Photochemical Reflectance index
ARI	Anthocyanin Reflectance Index
SIPI	Structure insensitive pigment index
mCAI	Modified chlorophyll absorption integral
PSSRa	Pigment specific simple ratio chlorophyll a
PSSRb	Pigment specific simple ratio chlorophyll b
PSSRc	Pigment specific simple ratio carotenoids
GM1	Gitelson and Merzlyak 1
GM2	Gitelson and Merzlyak 2
ZTM	Zarco-Tejada Miller
TCARI/OSAVI	Ratio of the Transformed Chlorophyll Absorption in Reflectance Index and Optimized Soil-Adjusted Vegetation Index

## Appendix A

**Table A1:** Results of SVIs in classifying different groups of spectral data acquired in the August acquisition campaign (Severity of infestation = 1).

Grapevine Variety	SVIs	Accuracy (%)	FNR (%)	FPR (%)	AUC
Marselan	NDVI	0.8710	0.1176	0.1429	0.9580
	PRI	0.9032	0.1111	0.0769	0.9160
	ARI	0.9032	0.1111	0.0769	0.9412
	SIPI	0.4516	0.5000	0.6667	0.4244
	mCAI	0.5806	0.3889	0.4615	0.5924
	PSSRa	0.5806	0.4091	0.4444	0.5126
	PSSRb	0.7742	0.1875	0.2667	0.7605
	PSSRc	0.4839	0.4762	0.6000	0.4244
	GM1	0.5484	0.4286	0.5000	0.5462
	GM2	0.8387	0.1250	0.2000	0.8992
	ZTM	0.8387	0.1250	0.2000	0.9244
	TCARI/OSAVI	0.8387	0.2000	0.0909	0.8193
Grenache	NDVI	0.9375	0.1053	0	0.9608
	PRI	0.9375	0.1053	0	0.9608
	ARI	0.9688	0	0.0625	1
	SIPI	0.8750	0.1579	0.0769	0.9529
	mCAI	0.8750	0.1905	0	0.8902
	PSSRa	0.5625	0.4286	0.4545	0.5020
	PSSRb	0.9375	0.1053	0	0.9451
	PSSRc	0.5938	0.4091	0.4000	0.6549
	GM1	0.6563	0.2500	0.4000	0.6471
	GM2	0.9375	0.1053	0	0.9529
	ZTM	0.9375	0.1053	0	0.9490
	TCARI/OSAVI	0.8750	0.1905	0	0.8314
Vermentino	NDVI	0.9063	0.1111	0.0714	0.9412
	PRI	0.9063	0.1111	0.0714	0.8863
	ARI	0.6875	0.3333	0.2727	0.6706
	SIPI	0.3438	0.5909	0.8000	0.2627
	mCAI	0.8438	0.2000	0.0833	0.8667
	PSSRa	0.5000	0.4737	0.5385	0.5373
	PSSRb	0.7813	0.2222	0.2143	0.8824
	PSSRc	0.6250	0.3684	0.3846	0.6000
	GM1	0.9063	0.1111	0.0714	0.9608
	GM2	0.8750	0.1176	0.1333	0.9255
	ZTM	0.9375	0.0588	0.0667	0.9490
	TCARI/OSAVI	0.8438	0.2000	0.0833	0.9490
Chardonnay	NDVI	0.9063	0.1111	0.0714	0.9059
	PRI	0.8438	0.2000	0.0833	0.9647
	ARI	0.6875	0.2941	0.3333	0.6784
	SIPI	0.3750	0.5652	0.7778	0.4314
	mCAI	0.8438	0.1667	0.1429	0.9137
	PSSRa	0.5938	0.4091	0.4000	0.5725
	PSSRb	0.8125	0.2105	0.1538	0.9490
	PSSRc	0.5313	0.4286	0.5000	0.5235
	GM1	0.8750	0.1176	0.1333	0.9020
	GM2	0.8750	0.1176	0.1333	0.9098
	ZTM	0.9063	0.1111	0.0714	0.9176
	TCARI/OSAVI	0.8438	0.1667	0.1429	0.8902
Red	NDVI	0.9206	0.1316	0	0.9545
	PRI	0.9365	0.1081	0	0.9576
	ARI	0.9524	0.0588	0.0345	0.9980
	SIPI	0.5556	0.4359	0.4583	0.6768
	mCAI	0.8254	0.2195	0.0455	0.8566
	PSSRa	0.5397	0.4118	0.4483	0.6000
	PSSRb	0.8413	0.2250	0.0870	0.8707
	PSSRc	0.4444	0.5405	0.6154	0.4313
	GM1	0.5079	0.3462	0.4324	0.5081
	GM2	0.9206	0.1316	0	0.8758
	ZTM	0.9206	0.1316	0	0.9414
	TCARI/OSAVI	0.6825	0.270	0.2308	0.7232
White	NDVI	0.6094	0.3667	0.4118	0.6393
	PRI	0.5313	0.4286	0.4091	0.4633
	ARI	0.8750	0.1795	0.0400	0.9013
	SIPI	0.8438	0.2051	0.0800	0.8700
	mCAI	0.8281	0.2439	0.0870	0.8123
	PSSRa	0.8125	0.2353	0.2333	0.8485
	PSSRb	0.7188	0.2941	0.3000	0.7185
	PSSRc	0.8906	0.0938	0.1250	0.9501
	GM1	0.9063	0.0882	0.0667	0.9550
	GM2	0.5781	0.4194	0.4545	0.5464
	ZTM	0.5781	0.3704	0.4324	0.6295
	TCARI/OSAVI	0.9219	0.1111	0.0357	0.9247
All	NDVI	0.6614	0.3043	0.2931	0.7251
	PRI	0.6063	0.4063	0.4286	0.5593
	ARI	0.8268	0.1884	0.1552	0.8970
	SIPI	0.8898	0.1549	0.0893	0.9134
	mCAI	0.8898	0.1471	0.1186	0.8841
	PSSRa	0.8661	0.1667	0.0909	0.8605
	PSSRb	0.5669	0.4179	0.4333	0.6022
	PSSRc	0.6378	0.2833	0.3284	0.6772
	GM1	0.8110	0.1940	0.1833	0.8658
	GM2	0.6614	0.3158	0.2549	0.7318
	ZTM	0.5984	0.4154	0.4355	0.6278
	TCARI/OSAVI	0.9213	0.1351	0.0189	0.9221

**Table A2:** Results of SVIs in classifying different groups of spectral data acquired in the September acquisition campaign (Severity of infestation = 2).

Grapevine Variety	SVIs	Accuracy (%)	FNR (%)	FPR (%)	AUC
Marselan	NDVI	0.7604	0.2400	0.2391	0.7234
	PRI	0.6250	0.3774	0.3721	0.6018
	ARI	0.9688	0.0400	0.0217	0.9805
	SIPI	0.7917	0.1778	0.2353	0.7972
	mCAI	0.9583	0.0213	0.0612	0.9440
	PSSRa	0.5104	0.4762	0.5000	0.4774
	PSSRb	0.6146	0.3571	0.4074	0.6331
	PSSRc	0.6667	0.3509	0.3077	0.6817
	GM1	0.9479	0.0769	0.0227	0.9709
	GM2	0.8021	0.2222	0.1667	0.7933
	ZTM	0.7292	0.2653	0.2766	0.7117
	TCARI/OSAVI	0.9375	0.0784	0.0444	0.9431
Grenache	NDVI	0.6543	0.3784	0.3182	0.6867
	PRI	0.6914	0.3000	0.3137	0.6873
	ARI	0.9753	0.0270	0.0227	0.9988
	SIPI	0.8395	0.2273	0.0811	0.8747
	mCAI	0.9383	0.0789	0.0465	0.9681
	PSSRa	0.7407	0.2500	0.2653	0.7377
	PSSRb	0.5679	0.4667	0.4118	0.5577
	PSSRc	0.7284	0.2222	0.2963	0.7506
	GM1	0.9383	0.1190	0	0.9969
	GM2	0.6790	0.3514	0.2955	0.7230
	ZTM	0.6296	0.4054	0.3409	0.6683
	TCARI/OSAVI	0.7901	0.2500	0.1707	0.8974
Vermentino	NDVI	0.9818	0	0.0323	0.9600
	PRI	0.9455	0.0769	0.0345	0.9613
	ARI	0.7455	0.2105	0.2778	0.8080
	SIPI	0.9273	0.0435	0.0938	0.9560
	mCAI	0.9818	0	0.0323	0.9613
	PSSRa	0.8364	0.2143	0.1111	0.8547
	PSSRb	0.9818	0	0.0323	0.9600
	PSSRc	0.6727	0.3333	0.3235	0.7320
	GM1	0.9455	0.0769	0.0345	0.9573
	GM2	0.9636	0.0400	0.0333	0.9600
	ZTM	0.9818	0	0.0323	0.9600
	TCARI/OSAVI	0.9636	0.0400	0.0333	0.9573
Chardonnay	NDVI	0.9773	0	0.0385	0.9537
	PRI	0.9545	0	0.0741	0.9474
	ARI	0.9318	0.0556	0.0769	0.9053
	SIPI	0.9318	0.1000	0.0417	0.9516
	mCAI	0.9773	0	0.0385	0.9474
	PSSRa	0.9091	0.1053	0.0800	0.9074
	PSSRb	0.9773	0	0.0385	0.9495
	PSSRc	0.7955	0.1429	0.2333	0.7853
	GM1	0.9773	0	0.0385	0.9495
	GM2	0.9773	0	0.0385	0.9516
	ZTM	0.9773	0	0.0385	0.9474
	TCARI/OSAVI	0.9773	0	0.0385	0.9579
Red	NDVI	0.6836	0.3011	0.2381	0.7092
	PRI	0.6554	0.3684	0.3663	0.6776
	ARI	0.9831	0.0233	0.0110	0.9991
	SIPI	0.8644	0.1573	0.1136	0.8377
	mCAI	0.9548	0.0357	0.0430	0.9510
	PSSRa	0.5989	0.4143	0.4112	0.5969
	PSSRb	0.5763	0.4096	0.3830	0.6084
	PSSRc	0.6893	0.3049	0.2947	0.7210
	GM1	0.9492	0.0957	0	0.9792
	GM2	0.7175	0.2929	0.1923	0.7063
	ZTM	0.6836	0.3889	0.3448	0.7196
	TCARI/OSAVI	0.8870	0.1538	0.0930	0.9633
White	NDVI	0.6465	0.4048	0.3158	0.6956
	PRI	0.5859	0.5667	0.4348	0.5199
	ARI	0.9899	0	0.0175	0.9788
	SIPI	0.9394	0.1064	0.0192	0.9767
	mCAI	0.9495	0.0238	0.0351	0.9784
	PSSRa	0.9495	0.0476	0.0526	0.9593
	PSSRb	0.8889	0.1304	0.0566	0.9435
	PSSRc	0.9798	0.0233	0.0179	0.9655
	GM1	0.9899	0	0.0175	0.9792
	GM2	0.7778	0.2955	0.2182	0.8223
	ZTM	0.5657	0.5000	0.4030	0.5793
	TCARI/OSAVI	0.9697	0.0233	0.0179	0.9950
All	NDVI	0.4674	0.6184	0.4900	0.4102
	PRI	0.8551	0.1944	0.0833	0.9300
	ARI	0.7029	0.3411	0.2857	0.7255
	SIPI	0.7138	0.3103	0.2938	0.7362
	mCAI	0.9420	0.0752	0.0280	0.9540
	PSSRa	0.6232	0.3962	0.3706	0.6362
	PSSRb	0.7899	0.2819	0.1575	0.8120
	PSSRc	0.8188	0.2365	0.1094	0.8512
	GM1	0.6775	0.3729	0.3354	0.6991
	GM2	0.5181	0.5049	0.4393	0.5523
	ZTM	0.5688	0.5208	0.4500	0.5637
	TCARI/OSAVI	0.8877	0.1298	0.0897	0.9160

**Table A3:** Results of SVIs in classifying different groups of spectral data acquired in the August and the September acquisition campaigns (Severity of infestation = 1 & 2).

Grapevine Variety	SVIs	Accuracy (%)	FNR (%)	FPR (%)	AUC
Marselan	NDVI	0.6929	0.3030	0.3115	0.7045
	PRI	0.7165	0.2899	0.2759	0.7318
	ARI	0.9370	0.0870	0.0345	0.9824
	SIPI	0.7402	0.2143	0.2958	0.7355
	mCAI	0.9213	0.1014	0.0517	0.8901
	PSSRa	0.5433	0.4407	0.4706	0.5330
	PSSRb	0.5118	0.4776	0.5000	0.5769
	PSSRc	0.5984	0.3833	0.4179	0.6150
	GM1	0.8583	0.2099	0.0217	0.9164
	GM2	0.7087	0.3056	0.2727	0.7419
	ZTM	0.7008	0.3151	0.2778	0.7655
	TCARI/OSAVI	0.8504	0.1714	0.1228	0.9002
Grenache	NDVI	0.7500	0.2381	0.2623	0.7846
	PRI	0.8226	0.1111	0.2286	0.7971
	ARI	0.9113	0.1370	0.0196	0.9753
	SIPI	0.8226	0.2083	0.1346	0.8521
	mCAI	0.9032	0.1286	0.0556	0.9318
	PSSRa	0.6048	0.3810	0.4098	0.5828
	PSSRb	0.6290	0.3676	0.3750	0.6547
	PSSRc	0.6532	0.3279	0.3651	0.6352
	GM1	0.8226	0.2558	0	0.8776
	GM2	0.7742	0.2429	0.2037	0.7893
	ZTM	0.7661	0.2388	0.2281	0.8089
	TCARI/OSAVI	0.7742	0.2857	0.1000	0.7620
Vermentino	NDVI	0.9080	0.0976	0.0870	0.9602
	PRI	0.8506	0.1316	0.1633	0.9014
	ARI	0.6207	0.3750	0.3818	0.5536
	SIPI	0.8161	0.2549	0.0833	0.7937
	mCAI	0.8391	0.2353	0.0556	0.8489
	PSSRa	0.6897	0.3600	0.2432	0.7312
	PSSRb	0.9080	0.0976	0.0870	0.9539
	PSSRc	0.6437	0.3438	0.3636	0.5938
	GM1	0.9540	0.0698	0.0227	0.9730
	GM2	0.9080	0.0976	0.0870	0.9491
	ZTM	0.9310	0.0513	0.0833	0.9655
	TCARI/OSAVI	0.9425	0.0909	0.0233	0.9459
Chardonnay	NDVI	0.9342	0.1053	0.0263	0.9659
	PRI	0.9342	0.0833	0.0500	0.9484
	ARI	0.8684	0.0968	0.1556	0.8725
	SIPI	0.8026	0.2917	0.0357	0.8474
	mCAI	0.9474	0.0811	0.0256	0.9589
	PSSRa	0.7237	0.3478	0.1667	0.7582
	PSSRb	0.9342	0.1053	0.0263	0.9247
	PSSRc	0.5658	0.4688	0.4091	0.5617
	GM1	0.9211	0.1081	0.0513	0.9568
	GM2	0.9605	0.0556	0.0250	0.9582
	ZTM	0.9605	0.0556	0.0250	0.9672
	TCARI/OSAVI	0.9342	0.1053	0.0263	0.9568
Red	NDVI	0.7331	0.2908	0.2545	0.7578
	PRI	0.6773	0.2719	0.3285	0.7174
	ARI	0.9323	0.1119	0.0093	0.9888
	SIPI	0.7968	0.2180	0.2034	0.8010
	mCAI	0.9203	0.1087	0.0442	0.9126
	PSSRa	0.5817	0.3760	0.3968	0.5854
	PSSRb	0.6574	0.3630	0.3621	0.6756
	PSSRc	0.5936	0.3740	0.3984	0.5963
	GM1	0.8486	0.2289	0	0.9032
	GM2	0.7809	0.2414	0.1698	0.7743
	ZTM	0.7331	0.2727	0.2222	0.7531
	TCARI/OSAVI	0.8088	0.2437	0.0769	0.8270
White	NDVI	0.6687	0.3333	0.3398	0.6903
	PRI	0.5644	0.4559	0.4000	0.5352
	ARI	0.9509	0.0864	0.0122	0.9433
	SIPI	0.9264	0.1395	0.0130	0.9517
	mCAI	0.9202	0.1125	0.0482	0.9385
	PSSRa	0.9202	0.1235	0.0488	0.9477
	PSSRb	0.8037	0.2527	0.0972	0.8503
	PSSRc	0.9202	0.1395	0.0130	0.9456
	GM1	0.9387	0.0988	0.0244	0.9658
	GM2	0.6503	0.3200	0.2727	0.6598
	ZTM	0.5951	0.4151	0.4000	0.5662
	TCARI/OSAVI	0.9387	0.1098	0.0247	0.9659
All	NDVI	0.5242	0.4805	0.4692	0.5060
	PRI	0.7850	0.2857	0.1818	0.7996
	ARI	0.7271	0.2786	0.2676	0.7574
	SIPI	0.7536	0.2764	0.2698	0.7741
	mCAI	0.8841	0.1696	0.0598	0.9184
	PSSRa	0.7222	0.2990	0.3000	0.7466
	PSSRb	0.7101	0.3103	0.2308	0.7353
	PSSRc	0.7343	0.3305	0.2472	0.7374
	GM1	0.6884	0.3368	0.3348	0.7130
	GM2	0.5459	0.4565	0.4435	0.5687
	ZTM	0.5580	0.4176	0.4221	0.5605
	TCARI/OSAVI	0.8744	0.1429	0.1078	0.8727
